# Temporal Modulation of HER2 Membrane Availability Increases Pertuzumab Uptake and Pretargeted Molecular Imaging of Gastric Tumors

**DOI:** 10.2967/jnumed.119.225813

**Published:** 2019-11

**Authors:** Patrícia M.R. Pereira, Komal Mandleywala, Ashwin Ragupathi, Lukas M. Carter, Jeroen A.C.M. Goos, Yelena Y. Janjigian, Jason S. Lewis

**Affiliations:** 1 Department of Radiology, Memorial Sloan Kettering Cancer Center, New York, New York; 2 Department of Medicine, Memorial Sloan Kettering Cancer Center, and Department of Medicine, Weill Cornell Medical College, New York, New York; 3 Molecular Pharmacology Program, Memorial Sloan Kettering Cancer Center, New York, New York; 4 Department of Pharmacology, Weill Cornell Medical College, New York, New York; 5 Department of Radiology, Weill Cornell Medical College, New York, New York; and; 6 Radiochemistry and Molecular Imaging Probes Core, Memorial Sloan Kettering Cancer Center, New York, New York

**Keywords:** HER2, pertuzumab, gastric tumors, lovastatin, pretargeting

## Abstract

Human epidermal growth factor receptor 2 (HER2) is used as a tumor biomarker and therapeutic target. Pertuzumab is an anti-HER2 antibody, and its binding to tumor cells requires HER2 to be present at the cell membrane. However, the cellular distribution of HER2 protein in gastric tumors is dynamic, and HER2 internalization decreases antibody binding to tumor cells. These features preclude the use of pretargeted strategies for molecular imaging and therapy. We explored the pharmacological modulation of HER2 endocytosis as a strategy to improve pertuzumab uptake in HER2-positive gastric tumors and allow the use of a pretargeted imaging approach. **Methods:** We conducted in vitro and in vivo studies with NCI-N87 gastric cancer cells to determine how HER2 endocytosis affects pertuzumab binding to tumor cells. Lovastatin, a clinically approved cholesterol-lowering drug, was used to modulate caveolae-mediated HER2 endocytosis. **Results:** Administration of lovastatin to NCI-N87 cancer cells resulted in significant accumulation of non-activated HER2 dimers at the cell surface. Pretreatment of NCI-N87 cells with lovastatin increased in vitro specific accumulation of membrane-bound ^89^Zr-labeled pertuzumab. Lovastatin-enhanced pertuzumab tumor uptake was also observed in NCI-N87 gastric cancer xenografts, allowing tumor detection as early as 4 h and high-contrast images at 48 h after tracer administration via PET. Temporal enhancement of HER2 membrane availability by lovastatin allowed imaging of cell surface HER2 with transcyclooctene-conjugated antibodies and ^18^F-labeled tetrazine. **Conclusion:** Temporal pharmacological modulation of membrane HER2 may be clinically relevant and exploitable for pretargeted molecular imaging and therapy in gastric tumors.

Members of the human epidermal growth factor receptor (HER) family (HER1, HER2, HER3, and HER4) are membrane receptor tyrosine kinases that in response to extracellular signals trigger downstream oncogenic signaling cascades (1). Aberrant cancer signaling—in pathways activated by HER family members—results from dysregulated receptor activation (mediated by receptor homo- and heterodimerization upon ligand binding, for example), receptor overexpression, or specific mutations (1–4). HER2 appears to have no direct ligand, and its indirect activation most likely is triggered by dimerization with other members of the HER family (1,5). Many cancers have amplification of the *HER2* gene or overexpression of HER2 protein (6,7). Therapies targeting HER2 have been very successful in the treatment of breast cancer (8,9), and monoclonal antibodies (trastuzumab and pertuzumab), antibody–drug conjugates (ado-trastuzumab emtansine), and tyrosine kinase inhibitors targeting both HER1 and HER2 (lapatinib) are clinically approved for the treatment of breast cancer.

HER2 is also a clinical biomarker and therapeutic target in patients with gastric tumors (3,10–16). Indeed, treating patients with HER2-positive metastatic gastric or gastroesophageal junction tumors with HER2-targeting trastuzumab plus chemotherapy has yielded improved overall survival compared with chemotherapy alone (10). Based on data supporting a synergetic effect of trastuzumab and pertuzumab (17), a dual HER2-blockade–plus–chemotherapy approach was tested in the JACOB trial. However, this combination did not significantly improve overall survival in patients with HER2-positive metastatic gastric or gastroesophageal junction cancer compared with placebo (18). Notably, a current limitation is that selection of patients for HER2-targeted trials is largely based on the assessment of HER2 status through immunohistochemistry of tumor biopsy specimens. This approach incompletely captures the cellular dynamics of HER2 and its heterogeneous expression in gastric tumors (15). The use of molecular imaging to evaluate the expression of receptors of the HER family is a promising strategy to improve patient selection for anti-HER therapies and monitor therapeutic response (19–23).

HER2 antibodies (trastuzumab or pertuzumab) radiolabeled with ^89^Zr have the potential to target and image HER2-positive tumors (21–24). However, clinical studies have reported that ^89^Zr-labeled antibodies do not always accumulate in HER2-positive tumors (25). Immunohistochemical staining of gastric tumors reveals nonuniform membrane expression of HER2 (15), which contributes to low accumulation of antibodies in these tumors (18,26,27). Moreover, endocytic trafficking mediates HER2 internalization and further reduces the availability of HER2 at the cell membrane, preventing binding with antibodies such as trastuzumab and pertuzumab and dampening their therapeutic efficacy (27–30).

The internalization of HER2 to the intracellular compartment not only decreases the ability of ^89^Zr-labeled antibodies to target HER2-positive tumors but also precludes the use of pretargeted strategies for molecular imaging and therapy (31–33). Pretargeting approaches have been developed to reduce radiation doses to healthy tissues associated with antibodies radiolabeled with long-lived radionuclides. The inverse electron demand Diels–Alder click chemistry–based in vivo pretargeting approach involves injection of a tumor-targeting antibody bearing a clickable handle, accumulation of the antibody in tumor over 24–72 h accompanied by clearance from blood, injection of a pharmacokinetically short-lived radioligand containing a clickable counterpart, and in vivo click between the radioligand and the membrane-accumulated antibody (31,32,34). Currently, the usefulness of such a pretargeted strategy for a rapidly internalizing antigen, such as HER2, is limited; antibody-mediated internalization of HER2 reduces the availability of the antibody and its associated clickable sites on the tumor for the incoming radioligand, which can bear an imaging or therapeutic radionuclide. HER2 is also a circulating antigen, and the injected antibody will not only target the antigen-expressing tumor tissue but also bind HER2 present in circulation. Therefore, in a pretargeted strategy, the small-molecule radiotracer will react with antibody-bound HER2 in circulation and increase the background-to-tumor ratios.

Caveolin-1 (CAV1), a protein present in cholesterol-rich structures at the cell membrane, mediates HER2 internalization and reduces HER2 availability at the cell membrane for binding with trastuzumab (27,35,36). We have previously shown that temporal modulation of CAV1 protein with the cholesterol-lowering drug lovastatin improved HER2 stability at the cell membrane and increased and accelerated tumoral uptake of ^89^Zr-labeled trastuzumab.

In this study, we explored the hypothesis that modulation of HER2 endocytosis would improve the accumulation of pertuzumab in HER2-positive gastric tumors and allow the use of a pretargeted approach in HER2-positive tumors.

## MATERIALS AND METHODS

### Cell Lines and Treatments

The HER2-positive/CAV1-positive gastric NCI-N87 and HER2-negative/CAV1-positive breast MDA-MB-231 human cancer cell lines were purchased from American Type Culture Collection (CRL 5822 and HTB-26) in 2014 and authenticated by the Memorial Sloan Kettering Cancer Center (MSK) Integrated Genomics Operation Core using short tandem repeat analysis. NCI-N87 and MDA-MB-231 cells were used within 15 passages and were confirmed to be *Mycoplasma*-free. NCI-N87 cells were maintained at 37°C in a humidified atmosphere at 5% CO_2_ in RPMI-1640 growth medium supplemented with 10% fetal calf serum, 2 mM l-glutamine, 10 mM 4-(2-hydroxyethyl)-1-piperazineethanesulfonic acid, 1 mM sodium pyruvate, a 4.5 g/L solution of glucose, a 1.5 g/L solution of NaHCO_3_, and a 100 unit/mL concentration of both penicillin and streptomycin. MDA-MB-231 cells were cultured in American Type Culture Collection–formulated Leibovitz L-15 medium supplemented with 10% fetal bovine serum.

NCI-N87 cells were incubated in medium containing 25 μM of the active form of lovastatin (Millipore) in medium for 4 h before addition of pertuzumab. Control experiments were performed by incubating NCI-N87 cancer cells in medium for 4 h before addition of pertuzumab.

### Western Blot Analysis

Whole-protein extracts from control or lovastatin-treated NCI-N87 cells were obtained by cell scraping at 4°C in radioimmunoprecipitation assay buffer (150 mM NaCl, 50 mM Tris-HCl, pH 7.5, 5 mM egtazic acid, 1% Triton X-100, 0.5% sodium deoxycholate, 0.1% sodium dodecyl sulfate, 2 mM phenylmethanesulfonyl, 2 mM iodoacetamide, and 1× protease inhibitor cocktail [C852A33; Roche]). After centrifugation at 16,000*g* for 10 min at 4°C, supernatants were used for protein quantification with the Pierce BCA Protein Assay Kit (23225; Thermo Fisher Scientific) and denatured with Laemmli buffer. After electrophoresis and transfer to nitrocellulose membranes (IB23001; Thermo Fisher Scientific), the blots were incubated in 5% (m/v) bovine serum albumin in Tris-buffered saline–polysorbate (9997S; Cell Signaling Technology) and probed with 1:20,000 mouse anti-β-actin (A1978; Sigma), 1:800 rabbit anti-HER2 (ab131490; Abcam), 1:800 rabbit anti–epidermal growth factor receptor (EGFR) (ab52894; Abcam), 1:500 rabbit antiphospho-p44/42 mitogen-activated protein kinase (MAPK) (9101S; Cell Signaling), 1:800 rabbit anti-p44/42 MAPK (9102S; Cell Signaling), and 1:500 mouse antiphosphotyrosine antibodies (05-321X; EMD Millipore). After washing, the membranes were incubated with 1:15,000 IRDye 800CW anti-rabbit (925-32211) or anti-mouse (925-32210) IgG (LI-COR Biosciences) and imaged on an Odyssey Infrared Imaging System (LI-COR Biosciences) followed by densitometric analysis using ImageJ software.

### Immunoprecipitation Assays

For immunoprecipitation experiments, total cellular protein (500 μL of radioimmunoprecipitation assay buffer containing 200 μg of protein) was incubated with 10 μg of primary antibody Neu (F-11) agarose conjugate (sc-7301; Santa Cruz Biotechnology) overnight at 4°C with gentle rotation. The pellet (containing the immunoprecipitated fraction) was collected by centrifugation at 1,000*g* for 30 s at 4°C and washed 3 times with radioimmunoprecipitation assay buffer before resuspension in Laemmli buffer.

### Pertuzumab DFO and TCO Conjugation

Clinical-grade pertuzumab (Perjeta; Genentech) was conjugated with *p*-isothiocyanatobenzyl-desferrioxamine (DFO-Bz-NCS) or transcyclooctene (TCO) as described previously (22). Briefly, a solution of pertuzumab (2.61 mg, 3.26 mg/mL) in phosphate-buffered saline (PBS), pH 7.4, was adjusted to pH 8.4 with 1 M NaHCO_3_ solution. Thirteen molar equivalents of the bifunctional chelate DFO-Bz-NCS (Macrocyclics, Inc, 10 mg/mL, 13.3 mM) in dimethyl sulfoxide were added. For conjugation with TCO, pertuzumab (3.40 mg, 3.40 mg/mL) in PBS (pH 7.4) was adjusted to pH 8.5 using 0.1 M Na_2_CO_3_. Pertuzumab was then reacted with 30 molar equivalents of TCO-*N*-hydroxysuccinimide (25.0 mg/mL, 94 mM prepared in *N,N*-dimethylformamide). The DFO or TCO conjugation reactions were prepared fresh before use by incubating the antibody with DFO-Bz-NCS or TCO-*N*-hydroxysuccinimide at 37°C for 90 min before purification with a PD10 desalting column (GE Healthcare). Antibody–DFO conjugate was used for radiolabeling with ^89^Zr, and antibody–TCO conjugates were used for pretargeted strategies.

### Radiolabeling of Pertuzumab and Tetrazines

^89^Zr was produced via proton bombardment of yttrium foil and isolated with high purity as [^89^Zr]Zr-oxalate at MSK using previously reported procedures (37). A neutralized solution of ^89^Zr-oxalate (37 MBq, pH 7.0–7.2) was added to pertuzumab-DFO (300 μg) in PBS at 37°C for 1 h before sequential purification with a Sephadex G-25 (PD10; GE Healthcare) desalting column and a regenerated cellulose centrifugal filter with a molecular weight cutoff of 50 kDa (Amicon; Millipore). [^89^Zr]Zr-pertuzumab with radiochemical purity of at least 95% as determined by instant thin-layer chromatography was used for in vitro and in vivo studies.

No-carrier-added [^18^F]fluoride was obtained via the ^18^O(p,n)^18^F nuclear reaction of 11-MeV protons on an ^18^O-enriched water target. [^18^F]AlF-NOTA-PEG_11_-tetrazine (Tz) was synthesized following procedures described previously (32) and used with the same radiochemical purity specification (>98% and molar activity = 55.5 MBq [1.5 mCi]/nmol) as determined by radio–high performance liquid chromatography (Supplemental Fig. 1; supplemental materials are available at http://jnm.snmjournals.org).

### Internalization Assays and Saturation-Binding Assays

For the internalization assays with [^89^Zr]Zr-DFO-pertuzumab, control or lovastatin-treated cells were incubated with cell culture medium in the presence of 1 μM [^89^Zr]Zr-DFO-pertuzumab for 90 min at 37°C. Media containing non–cell-bound radiotracer was removed, and the cells were washed twice with PBS. Cell surface–bound radiotracer was collected by incubation at 4°C for 5 min in 0.2 M glycine buffer containing 0.15 M NaCl and 4 M urea at pH 2.5. The internalized fraction was obtained after cell lysis with 1 M NaOH. The 3 fractions were measured for radioactivity on a γ-counter calibrated for ^89^Zr.

For the saturation-binding assays, cells were incubated with ^89^Zr-labeled pertuzumab (0–256 nM) in PBS (pH 7.5) containing 1% (m/v) human serum albumin (Sigma) and 1% (m/v) sodium azide (Acros Organics) for 2 h at 4°C. Unbound radioactivity was removed, and cells were washed 3 times with PBS. The cells were solubilized in 100 mM NaOH and were recovered, and the total cell-bound radioactivity was measured on a γ-counter calibrated for ^89^Zr. Total binding was plotted versus the concentration of ^89^Zr-pertuzumab; the data were fit via nonlinear regression with a 1-site binding model in GraphPad Prism 7.00 to determine B_max_. The nonspecific component was subtracted from the total binding to generate specific binding curves (Supplemental Fig. 2; supplemental materials are available at http://jnm.snmjournals.org).

### In Vitro Blocking Experiments

Blocking experiments were performed by incubating cells with ^89^Zr-labeled pertuzumab in the presence of a 30-fold excess of trastuzumab or pertuzumab.

### Tumor Xenografts

Experiments on animals were conducted according to the guidelines approved by the Research Animal Resource Center and Institutional Animal Care and Use Committee at MSK. The first author (Pereira) has a Category C accreditation for animal research from the Federation of European Laboratory Animal Science. We adhere to the Animal Research: Reporting of In Vivo Experiments guidelines and to the guidelines for the welfare and use of animals in cancer research. Eight- to 10-wk-old *nu/nu* female mice (Charles River Laboratories) were injected subcutaneously on the right shoulder with 5 million NCI-N87 cells in a 150-μL cell suspension of a 1:1 (v/v) mixture of medium with reconstituted basement membrane (BD Matrigel, BD Biosciences). MDA-MB-231 cells were implanted orthotopically (5 million cells) in the lower right mammary fat pad in 50 μL of 1:1 Matrigel (BD Biosciences). The mice were housed in type II polycarbonate cages, fed a sterilized standard laboratory diet, and received sterile water ad libitum. The animals were housed at approximately 22°C and 60% relative humidity, and a 12-h light, 12-h dark cycle was maintained. After arrival, all mice were allowed to acclimate to the facility’s laboratory conditions for 1 wk before experimentation.

The tumor volume (*V*, mm^3^) was estimated by external vernier caliper measurements of the longest axis, *a* (mm), and the axis perpendicular to the longest axis, *b* (mm). The tumors were assumed to be spheroidal, and the volume was calculated using the equation *V* = (4π/3) × (*a*/2)^2^ × (*b*/2). When the volume of xenografts reached approximately 100 mm^3^, the mice were randomized into groups and treatments were initiated (*n* = 5 mice per group for biodistribution and *n* = 3 mice per group for PET imaging).

For the imaging experiments with ^89^Zr-labeled pertuzumab, the mice were divided into 2 groups: group 1, which received PBS orally 12 h before and at the same time as the tail vein injection of [^89^Zr]Zr-DFO-pertuzumab (4.44–5.18 MBq, 42–49 μg of protein), and group 2, which received lovastatin orally (8.3 mg/kg of mouse) 12 h before and at the same time as the tail vein injection of [^89^Zr]Zr-DFO-pertuzumab.

### In Vivo Blocking Experiments

Blocking experiments were performed on tumor-bearing mice injected with ^89^Zr-labeled pertuzumab in the presence of a 40-fold excess of trastuzumab or pertuzumab.

### Pretargeting

For the pretargeting experiments, lovastatin (8.3 mg/kg of mice) was orally administered 12 h before and at the same time as the tail vein injection of pertuzumab-TCO or trastuzumab-TCO (0.42 nmol). At 24 h after injection of antibody, [^18^F]AlF-NOTA-PEG_11_-Tz (14.73–16.54 MBq, 0.83–1.01 nmol) was injected via the tail vein.

### Acute Biodistribution Studies

Acute biodistribution studies were performed according to previously reported methods (32).

### PET and PET/CT Imaging

Imaging experiments were conducted on a microPET Focus 120 scanner (Concorde Microsystems) or an Inveon PET/CT scanner (Siemens). The mice were anesthetized by inhalation of 1.5%–2% isoflurane (Baxter Health care) in an oxygen gas mixture 10 min before the PET images were recorded. PET data for each group (*n* = 3) were recorded, with mice under isoflurane anesthesia (1.5%–2%), in list mode at 4, 8, 24, and 48 h after intravenous injection of [^89^Zr]Zr-DFO-pertuzumab. PET/CT data for each group (*n* = 3) were acquired 0.5, 1.5, and 4 h after injection of ^18^F-labeled Tz. List-mode emission data were sorted into 2-dimensional sinograms via Fourier rebinning; data were normalized to correct for nonuniform detector response, dead-time count losses, and positron branching ratio, but no attenuation, scatter, or partial-volume averaging corrections were applied. A 3-dimensional ordered-subset expectation maximization/maximum a posteriori (2 ordered-subset expectation maximization iterations/18 maximum a posteriori iterations) was used for reconstruction, and each reconstructed image was smoothed by convolution with a gaussian filter kernel of 1.5 mm in full width at half maximum to reduce noise. All images were visualized in AMIDE 1.0.4 software (http://amide.sourceforge.net).

### Statistical Analysis

Data are expressed as mean ± SEM. Groups were compared using the Student *t* test.

## RESULTS

### Lovastatin Increases HER2 Dimerization Without Altering HER2 Phosphorylation or Activation

**FIGURE 1.**
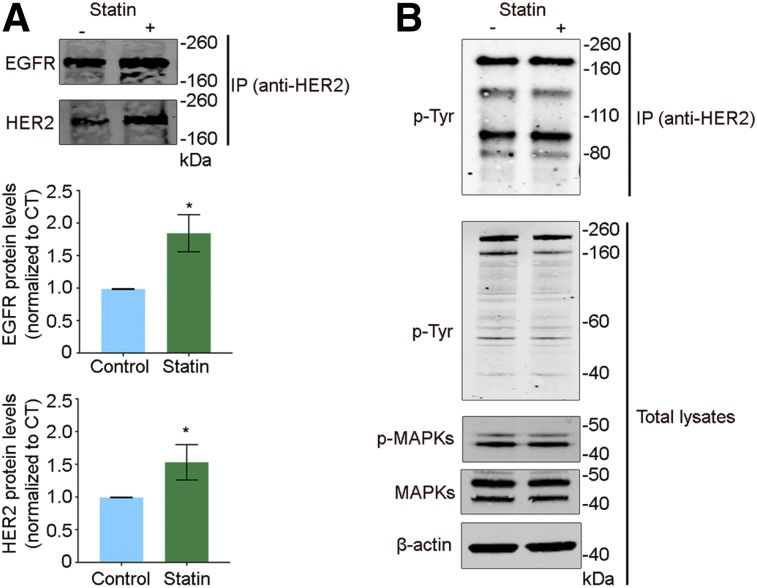
Lovastatin treatment increases HER2–HER2 and HER2–EGFR non-activated dimers in gastric cancer cells. Western blots are shown for EGFR, HER2, phosphorylated tyrosine (p-Tyr), phosphorylated MAPKs (p-MAPKs), and total MAPKs from total cell extracts or extracts obtained after immunoprecipitation (IP) with anti-HER2 antibody. Untreated NCI-N87 gastric cancer cells served as control. NCI-N87 cells were incubated with 25 μM lovastatin for 4 h. β-actin was used as loading control. Western blot quantifications of EGFR and HER2 (normalized to control) are represented as mean ± SEM (*P < 0.05 based on Student *t* test). Experiment was repeated 3 times.

Temporal modulation of CAV1 with the cholesterol-lowering drug lovastatin increases HER2 availability at the cell membrane to enhance the binding of trastuzumab to gastric cancer cells (27). Our previous work showed an increase in HER2 half-life at the cell membrane of NCI-N87 gastric cancer cells. Based on these findings, we investigated HER2 dimerization on treatment of these cells with lovastatin. Homodimerization and heterodimerization of HER2 are known to lead to autophosphorylation and further induction of downstream pro-oncogenic signaling pathways (4,5,38). Treatment of NCI-N87 gastric cancer cells with 25 μM of the active form of lovastatin for 4 h significantly enhanced the formation of HER2–HER2 homodimers (1.7 ± 0.3, *n* = 3) and HER2–EGFR heterodimers (2.0 ± 0.3, *n* = 3) in NCI-N87 gastric cancer cells (Fig. 1A). We did not detect HER2–HER3 heterodimers (data not shown), possibly because of the low levels of HER3 in NCI-N87 gastric cancer cells (17) or the fact that our experiments were not performed in the presence of a ligand (39). Although lovastatin treatment increased HER2–HER2 and HER2–EGFR dimers, we did not detect alterations in HER2 phosphorylation or in phosphotyrosine-containing proteins (Fig. 1B). Additional Western blot analyses revealed that lovastatin treatment did not impact the downstream MAPK pathway (Fig. 1B). Collectively, our results are consistent with an increase in HER2 availability at the cell membrane on treatment with lovastatin and the formation of non-activated dimerized HER2 receptors.

### Lovastatin Improves Molecular Imaging with ^89^Zr-Labeled Pertuzumab

**FIGURE 2.**
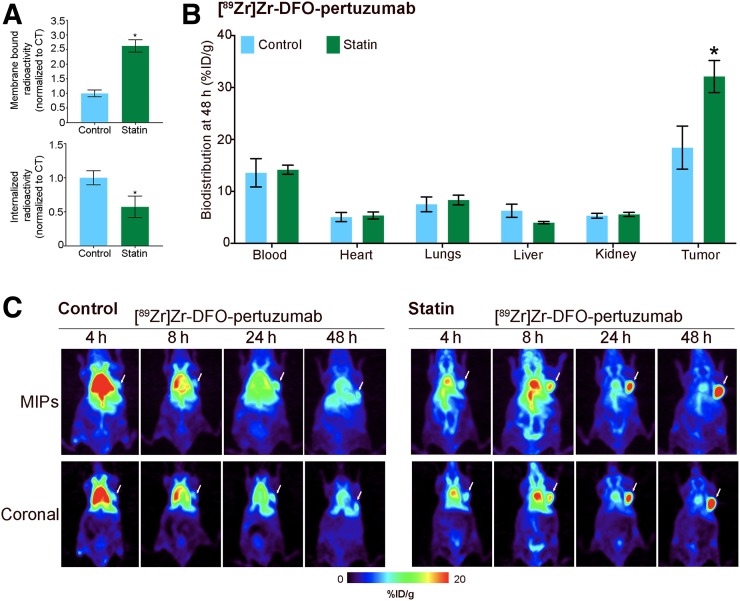
Lovastatin treatment increases in vitro membrane-bound pertuzumab and in vivo tumors’ avidity for pertuzumab. (A) Membrane-bound and internalized [^89^Zr]Zr-DFO-pertuzumab after treatment with lovastatin in NCI-N87 cancer cells. NCI-N87 untreated cells served as control. NCI-N87 cancer cells were incubated with 25 μM lovastatin for 4 h before addition of 1 μM ^89^Zr-labeled pertuzumab for 1.5 h. Data represent mean ± SEM (*n* = 4 experiments, *P < 0.05 based on Student *t* test). (B and C) Biodistribution (B) and representative maximum-intensity-projection images (MIPs) and coronal PET images (C) of [^89^Zr]Zr-DFO-pertuzumab in athymic nude mice bearing subcutaneous NCI-N87 gastric tumors treated with lovastatin. Lovastatin (8.3 mg/kg of mouse) was orally administered 12 h before and at same time as tail vein injection of [^89^Zr]Zr-DFO-pertuzumab (4.44–5.18 MBq, 42–49 μg of protein). Control mice received oral saline instead of lovastatin. Biodistribution data represent mean ± SEM (*n* = 5 mice per group, *P < 0.05 based on Student *t* test).

Prompted by our previous studies demonstrating that lovastatin treatment increases the avidity of HER2-positive tumors for trastuzumab (27), we performed in vitro and in vivo studies with ^89^Zr-labeled pertuzumab. Pertuzumab, a HER2 heterodimerization inhibitor, binds to the dimerization hairpin on the extracellular domain II of HER2 (40). Given our in vitro findings (Fig. 1A), we expected that changes in non-activated membrane HER2 dimers upon treatment with lovastatin would affect the ability of pertuzumab to bind tumor cells. Cellular fractionation experiments revealed a significant increase in membrane-associated ^89^Zr-labeled pertuzumab in cells pretreated with lovastatin compared with the control group (Fig. 2A). The increase in membrane-bound pertuzumab was accompanied by a significant decrease in the amount of internalized radioactivity (Fig. 2A). Additional competitive radioligand saturation-binding assays confirmed that lovastatin treatment increases pertuzumab binding to membrane HER2 in NCI-N87 cells (B_max_; Supplemental Fig. 2).

In vivo studies were then performed to further investigate the ability of lovastatin to increase pertuzumab binding to gastric tumors. Oral administration of lovastatin (8.3 mg/kg) to athymic mice bearing subcutaneous NCI-N87 gastric tumors was performed 12 h before and at the time of ^89^Zr-labeled pertuzumab injection [^89^Zr]Zr-DFO-pertuzumab (4.44–5.18 MBq, 42–49 μg of protein) (27). The control cohort received saline orally instead of lovastatin. PET imaging and biodistribution studies were performed 4, 8, 24, and 48 h after injection of ^89^Zr-labeled pertuzumab. An increase in ^89^Zr-labeled pertuzumab uptake over time was observed in both groups (Figs. 2B and 2C). In the lovastatin-treated group, tumors could be delineated at 4 h after injection of pertuzumab. Biodistribution studies revealed that at 48 h after injection of pertuzumab, tumor uptake of the radiolabeled analog of pertuzumab was higher in lovastatin-treated mice than in control mice (Fig. 2B, Supplemental Figs. 3 and 4). Tumors from control mice had an uptake of 18.4 ± 7.2 percentage injected dose per gram (%ID/g) (*n* = 5), whereas tumors from lovastatin-treated mice yielded a tumor uptake of 32.1 ± 6.9 %ID/g (*n* = 5). Control experiments in HER2-positive/CAV1-positive NCI-N87 gastric xenografts demonstrated that the tumor uptake of a radiolabeled isotype control IgG was significantly low and comparable in both saline-treated mice as well as those treated with lovastatin (27). Additional control studies in a HER2-negative/CAV1-positive MDA-MB-231 orthotopic mammary fat pad model demonstrated that ^89^Zr-labeled pertuzumab accumulation does not increase on lovastatin treatment in a HER2-negative tumor model (Supplemental Figs. 5 and 6).

**FIGURE 3.**
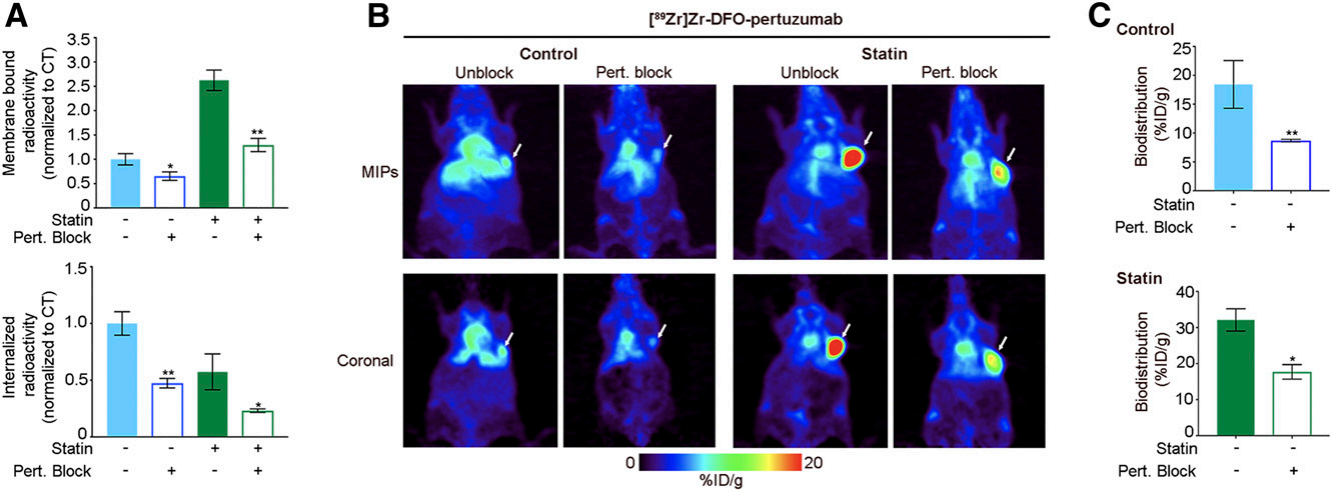
Lovastatin-mediated increase in membrane-bound pertuzumab is blocked with excess of pertuzumab. (A) Membrane-bound and internalized [^89^Zr]Zr-DFO-pertuzumab in presence of excess of unlabeled pertuzumab after lovastatin treatment in NCI-N87 cells. Blocking experiments were performed by incubation with ^89^Zr-labeled pertuzumab in presence of 30-fold excess of pertuzumab. Untreated NCI-N87 cells served as control. NCI-N87 cells were incubated with 25 μM lovastatin for 4 h before addition of 1 μM ^89^Zr-labeled pertuzumab and incubation for 1.5 h. Data represent mean ± SEM (*n* = 4 experiments, *P < 0.05 and **P < 0.01 based on Student *t* test). (B and C) Representative maximum-intensity-projection images (MIPs) and coronal PET images (B) and tumor uptake (at 48 h after injection of ^89^Zr-labeled pertuzumab) (C) in athymic nude mice bearing subcutaneous NCI-N87 gastric tumors with and without blocking with unlabeled pertuzumab. Lovastatin (8.3 mg/kg) was orally administered 12 h before and at same time as tail vein injection of [^89^Zr]Zr-DFO-pertuzumab (4.44–5.18 MBq, 42–49 μg of protein). Control mice received oral saline. Blocking experiments were performed by administration of ^89^Zr-labeled pertuzumab in presence of 40-fold excess of pertuzumab. Data represent mean ± SEM (*n* = 5 mice per group, *P < 0.05 and **P < 0.01 based on Student *t* test).

Additional in vitro studies demonstrated that lovastatin-mediated increases in membrane-bound pertuzumab could be blocked with a 30-fold excess of pertuzumab in NCI-N87 cells (Fig. 3A). In addition, ^89^Zr-labeled pertuzumab was injected into athymic nude mice bearing subcutaneous NCI-N87 gastric tumors that were blocked with a 40-fold excess of unlabeled pertuzumab. Immuno-PET images and ex vivo biodistribution acquired at 48 h after injection of antigen-blocked mice showed a significant reduction in tumor uptake in control mice (8.7 ± 0.4 %ID/g, *n* = 5) and lovastatin-treated mice (17.7 ± 3.5 %ID/g, *n* = 5) (Figs. 3B and 3C; Supplemental Figs. 3 and 4). Taken together, these studies support the potential of temporal lovastatin treatment to enhance the avidity of HER2-positive tumors for pertuzumab.

### Trastuzumab-Receptor Blockade Increases Internalization of ^89^Zr-Labeled Pertuzumab

**FIGURE 4.**
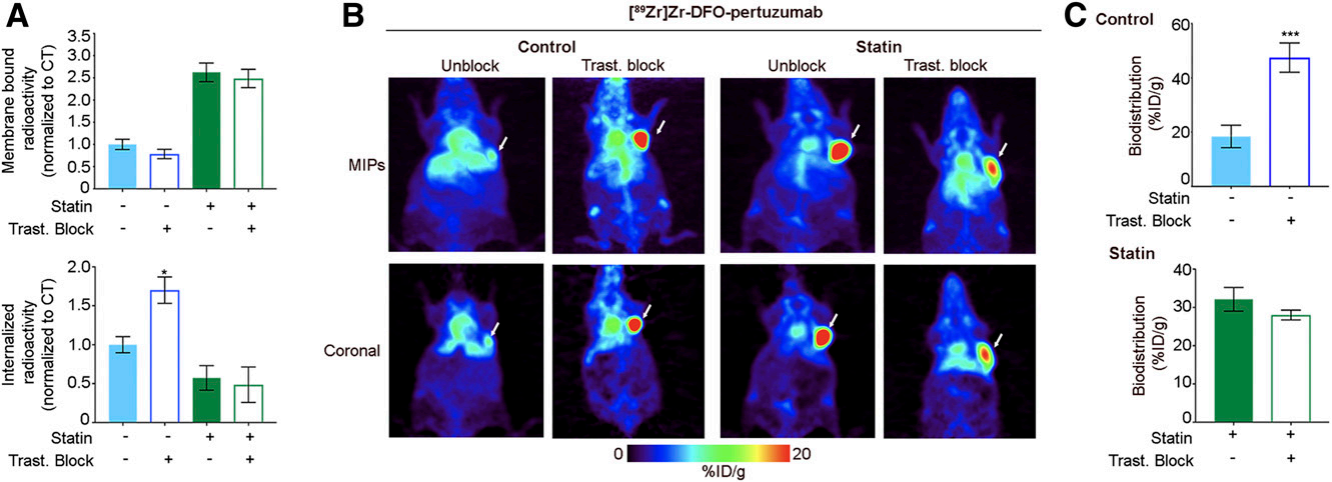
Trastuzumab-receptor blockade increases internalization of ^89^Zr-labeled pertuzumab. (A) Membrane-bound and internalized [^89^Zr]Zr-DFO-pertuzumab in presence of trastuzumab in NCI-N87 cancer cells with or without lovastatin treatment. Blocking experiments were performed by incubation with ^89^Zr-labeled pertuzumab in presence of 30-fold excess of trastuzumab. NCI-N87 untreated cells served as control. NCI-N87 cancer cells were incubated with 25 μM lovastatin for 4 h before addition of 1 μM ^89^Zr-labeled pertuzumab for 1.5 h. Data represent mean ± SEM (*n* = 4 experiments, *P < 0.05 based on Student *t* test). (B and C) Representative coronal PET images (B) and tumor uptake (at 48 h after injection of ^89^Zr-labeled pertuzumab) (C) in athymic nude mice bearing subcutaneous NCI-N87 gastric tumors with and without blocking with unlabeled trastuzumab. Lovastatin (8.3 mg/kg of mice) was orally administered 12 h before and at same time as tail vein injection of [^89^Zr]Zr-DFO-pertuzumab (4.44–5.18 MBq, 42–49 μg of protein). Control mice received oral saline. Blocking experiments were performed by administration of ^89^Zr-labeled pertuzumab in presence of 40-fold excess of trastuzumab. Data represent mean ± SEM (*n* = 5 mice per group, ***P < 0.001 based on Student *t* test). MIP = maximum-intensity projection.

We also investigated the effects of unlabeled trastuzumab in modulating the tumor-targeting ability of radiolabeled pertuzumab in both control and lovastatin-treated gastric tumor cells and xenograft mice. Trastuzumab binds to the extracellular domain of HER2 at a different epitope than pertuzumab (41), and previous preclinical studies have demonstrated that pertuzumab affinity is enhanced in the presence of trastuzumab (24,42). We explored whether the increase in pertuzumab binding in the presence of trastuzumab was due to alterations in receptor endocytic trafficking. Cellular fractionation experiments were performed on control and lovastatin-treated cells incubated with ^89^Zr-pertuzumab in the presence and absence of a 30-fold excess of unlabeled trastuzumab. In lovastatin-treated cells, excess trastuzumab did not induce significant alterations in the amount of membrane or internalized radiolabeled pertuzumab (Fig. 4A). In comparison, the amount of ^89^Zr-labeled pertuzumab in the intracellular fraction was 1.7-fold higher in NCI-N87 control cells incubated with an excess of trastuzumab.

To further these findings, in vivo studies with unlabeled trastuzumab were conducted on both control and lovastatin-treated xenograft mice. The immuno-PET images and ex vivo biodistribution in mice pretreated with lovastatin at 48 h after injection of the radiotracer were very similar in the presence and absence of trastuzumab (Fig. 4B; Supplemental Figs. 3 and 4). For the control mice, tumor accumulation of ^89^Zr-labeled pertuzumab was significantly increased in the presence of trastuzumab (47.5 ± 9.3 %ID/g, *n* = 5). ^89^Zr is described as a residualizing radiometal; the radiometal is trapped inside the cell after radioconstruct internalization and proteolytic degradation until it is externalized relatively slowly. Therefore, tumor uptake at 48 h after injection is a result of not only receptor-bound but also internalized radioactivity. These results indicate that the increase in the amount of internalized ^89^Zr-labeled pertuzumab is a result of trastuzumab-mediated HER2 internalization, an effect that can be temporally modulated by treatment with lovastatin.

### Lovastatin Allows ^18^F-Based Pretargeted PET Imaging of HER2-Positive Tumors

**FIGURE 5.**
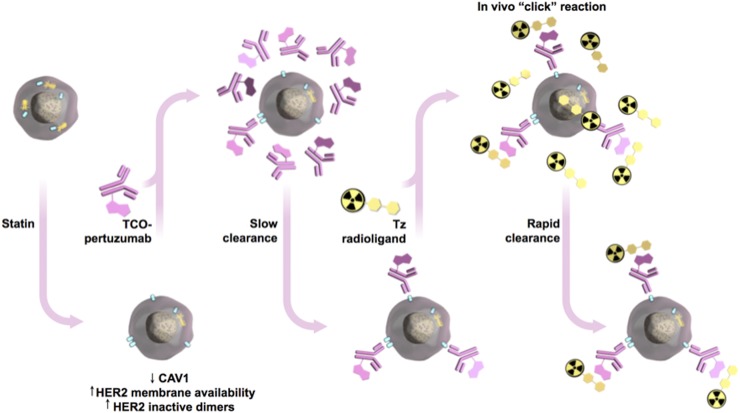
Schematic of pretargeting approach to image gastric tumors with pertuzumab in presence of lovastatin. Lovastatin depletes CAV1, increasing HER2 membrane availability and HER2 inactive dimers for binding pertuzumab. TCO-labeled pertuzumab (slow pharmacokinetics) is injected days ahead of administering radiolabeled small molecule. Then, only hours before imaging, administered radiolabeled small molecule travels through blood rapidly, either clicking with TCO-labeled antibody or quickly clearing from patient. HER2 is represented in light blue and CAV1 in yellow.

**FIGURE 6.**
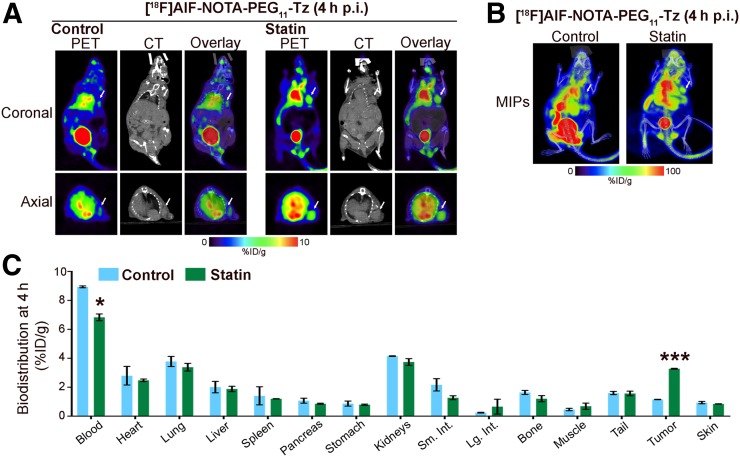
Pretreatment of gastric tumor cells with lovastatin improves pretargeted molecular imaging. Shown are representative coronal PET images (A), maximum-intensity-projection images (MIPs) (B), and biodistribution (C) at 4 h after injection of [^18^F]AlF-NOTA-PEG_11_-Tz in athymic nude mice bearing subcutaneous gastric tumors. Lovastatin (8.3 mg/kg of mouse) was orally administered 12 h before and at same time as tail vein injection of pertuzumab-TCO. Mice were administered pertuzumab-TCO (0.42 nmol) 24 h before injection of ^18^F-labeled tracer (14.73–16.54 MBq, 0.83 nmol) via tail vein. Data represent mean ± SEM (*n* = 5 experiments).

The rapid internalization of HER2 on antibody binding is incompatible with pretargeted molecular imaging since it does not allow sufficient membrane-bound anti-HER2 antibody to be available for binding a radiolabeled small molecule. We hypothesized that our strategy of temporal modulation of the membrane bioavailability and stability of HER2 by treatment with lovastatin could extend the benefits of pretargeted immuno-PET to this class of rapidly internalizing tumor-associated antigens (Fig. 5). On the basis of our data supporting lovastatin stabilization of HER2 at the cell surface, we predicted that antibody-mediated HER2 internalization would be lower in the presence of lovastatin than in control mice, which will enhance pertuzumab binding to membranous HER2. As such, a radiolabeled small molecule (in our case, [^18^F]AlF-NOTA-PEG_11_-Tz) would be able to conjugate bioorthogonally to TCO-labeled antibody (trastuzumab or pertuzumab) bound to HER2. To explore this, we performed in vivo pretargeting experiments on nude mice bearing subcutaneous NCI-N87 gastric tumors. Based on our Western blot analysis showing that in vivo reduction of CAV1 protein occurs between 12 and 48 h after administration of lovastatin (Supplemental Fig. 7), our pretargeting experiments were conducted with a 24-h interval between the injection of trastuzumab-TCO or pertuzumab-TCO and radiolabeled tetrazine (Tz). In lovastatin-treated mice, ^18^F-PET images at 4 h after injection of radiolabeled Tz clearly delineated HER2-positive tumors (Fig. 6; Supplemental Table 4). A pretargeting approach with trastuzumab-TCO and [^18^F]AlF-NOTA-PEG_11_-Tz (Supplemental Fig. 8) demonstrated increasing tumor uptake over time (1.86 ± 0.83 %ID/g at 30 min, 3.08 ± 0.50 %ID/g at 1.5 h, and 4.20 ± 1.03 %ID/g at 4 h) and decreasing blood radioactivity over time, from 6.01 ± 0.41 %ID/g at 30 min to 3.44 ± 0.44 %ID/g at 4 h, in mice pretreated with lovastatin.

In pretargeting approaches using pertuzumab-TCO (Fig. 6), biodistribution data at 4 h after injection of the radioligand revealed higher tumor uptake in lovastatin-treated mice ([^18^F]AlF-NOTA-PEG_11_-Tz, 3.30 ± 0.04 %ID/g) than in control mice ([^18^F]AlF-NOTA-PEG_11_-Tz, 1.20 ± 0.02 %ID/g). Although tumor-to-blood ratios were higher after treatment with lovastatin, the results suggest that after 24 h not all pertuzumab-TCO had been cleared from the blood. Previous studies have demonstrated that an increase in the administration time between antibody-TCO and radiolabeled Tz improves the tumor-to-background contrast in pretargeting strategies (31). In our study, such a strategy was not feasible because CAV1 modulation with lovastatin is a transient effect, and CAV1 levels at 48 h after administration of lovastatin are similar to those found in control tumors (Supplemental Fig. 7). These results suggest the potential of pretargeted molecular imaging with anti-HER2 antibodies in HER2-positive tumors depleted of CAV1 protein.

## DISCUSSION

Patient selection for therapy with anti-HER2 antibodies has been primarily based on HER2 positivity as assessed by total protein levels or gene amplification in tumor biopsy samples. This approach may not be reliable for gastric tumors, which display heterogeneous HER2 expression, and may contribute to the observed poor clinical response to pertuzumab/trastuzumab combinations in patients with gastric cancers (19). Few studies have attempted to determine the extent to which receptor membrane dynamics affect HER2 detection and anti-HER2 antibody therapy. In previous work, we identified an inverse relationship between the expression levels of CAV1 and the presence of HER2 protein at the tumor cell membrane (27). We showed that pharmacological modulation of CAV1 with lovastatin increases membrane HER2 availability for binding of trastuzumab in breast and gastric cancer cells. The use of lovastatin as a pharmacological modulator of CAV1 increased the uptake of trastuzumab even in tumors with non-predominant HER2 membrane staining.

In this study, we extended our prior work and gained further insights into how lovastatin affects HER2 stability at the cell membrane and binding of anti-HER2 antibodies to target cells. We found that lovastatin-induced accumulation of membrane HER2 is associated with an increase in the amount of non-activated HER2 dimers (Fig. 1). We used molecular imaging to show that lovastatin increases membrane (in vitro) and tumor (in vivo) accumulation of pertuzumab (Figs. 2–4), a humanized HER2-targeted antibody that binds to the dimerization domain of HER2. Finally, using a pretargeted molecular imaging approach in NCI-N87 xenograft mice pretreated with lovastatin, we demonstrated that ^18^F-labeled Tz could delineate HER2-positive tumors despite the biologic characteristics of HER2 as a circulating antigen and internalization of the TCO-conjugated antibody (Figs. 5 and 6).

The cytoplasmic catalytic function of receptor tyrosine kinases is activated on binding of a specific ligand to the monomeric receptor. This process induces receptor dimerization and autophosphorylation of tyrosine residues that in turn activates downstream signaling cascades (1,5). The PI3K-activated AKT pathway and p70S6K/p85S6K pathway are downstream signaling pathways activated by HER dimerization, and the Ras- and Shc-activated MAPK pathway is a target of all HER ligands (1). Although there are no known ligands that bind to HER2, the receptor is activated on dimerization with other members of the HER family (1,5). In gastric cancer, HER2 and HER3 overexpression and dimerization are associated with poor survival (43). One mechanism that decreases downstream signaling mediated by the HER family involves ligand-mediated receptor endocytosis (1). Preclinical studies have demonstrated that HER2 protein levels at the cell membrane are downregulated after treatment with trastuzumab because of an antibody-mediated internalization process (1). CAV1 depletion leads to HER2 accumulation at the cell membrane and enhanced HER2–HER2 and HER2–EGFR dimer formation (Fig. 1). Furthermore, we found that the increase in HER2–HER2 and HER2–EGFR dimers in response to lovastatin occurs without consequent receptor phosphorylation or activation of the MAPK pathway. Lovastatin increases HER2 dimerization without an alteration in HER2 downstream oncogenic signaling, plausibly because of the fact that our lovastatin pharmacological approach induced a transient effect on CAV1 depletion and HER2 availability at the cell membrane. Further studies are necessary to determine changes in CAV1 protein levels and HER2 membrane availability and downstream oncogenic signaling after treatment with single, fractionated, and prolonged doses of lovastatin.

The presence of high levels of HER2 dimers on the cell surface could serve as an antibody trap to enhance binding with anti-HER2 antibodies. Pertuzumab is a HER2-targeted humanized antibody, and it inhibits HER2 dimerization after binding domain II of the HER2 protein (40). Pertuzumab is used clinically for the treatment of metastatic HER2-overexpressing breast cancer. Radiolabeled pertuzumab also enables preclinical (24) and clinical (22) noninvasive, antibody-directed imaging of HER2-positive breast cancer. Molecular imaging with ^89^Zr-labeled pertuzumab is not possible at early time points because of its accumulation in the liver and blood (22). Indeed, PET imaging of BT474 breast cancer xenografts using ^89^Zr-labeled pertuzumab was possible only 120–168 h after administration of the radiotracer (24). The clinical use of ^89^Zr-labeled pertuzumab required that breast cancer patients return to the clinic for a PET scan 5–8 d after radiotracer administration (22). Our approach using lovastatin allowed tumor delineation at early time points and enhanced contrast 48 h after tracer injection; this strategy could improve the use of anti-HER2 antibodies for molecular imaging. The ability of lovastatin in depleting CAV1 protein in a transient manner is also a potential pharmacological strategy in molecular imaging with antibody fragments and engineered variants because these biomolecules exhibit faster accumulation in the tumor tissue than do fully intact antibodies.

Trastuzumab is an anti-HER2 humanized antibody that, in combination with chemotherapy, is a first-line treatment for patients with gastric tumors (10). However, trastuzumab has not been established as a second-line treatment of advanced gastric cancer (44). In metastatic breast tumors, dual HER2 blockade using pertuzumab and trastuzumab is more effective in the inhibition of HER2 signaling than trastuzumab alone (45). These agents are thought to have complementary activities: trastuzumab binds to domain IV of HER2 (41) and inhibits ligand-independent signaling (46), whereas pertuzumab binds to the HER2 dimerization domain II (40) to inhibit ligand-dependent signaling (47). Preclinical studies have shown that the combination of trastuzumab and pertuzumab is more effective in HER2-positive human gastric cancer xenografts than either antibody alone (17). However, in recent clinical trials, the combination of pertuzumab and trastuzumab did not improve outcomes in patients with metastatic or advanced gastric tumors (18). This result may be due, in part, to limitations in the current strategies to select patients with HER2-positive gastric tumors. Patient selection for HER2-targeted therapies is currently based on tumor-specific amplification of the *HER2* gene (positive fluorescence in situ hybridization) or overexpression of the HER2 protein (immunohistochemistry score of 3+) (10). Patient selection based on fluorescence in situ hybridization and immunohistochemistry has limitations when evaluating a heterogeneous target such as HER2. In comparison to breast tumors, immunohistochemistry in gastric tumors shows that HER2 exhibits heterogeneous (15) and incomplete membrane staining (48). These studies support the need for improved strategies to identify patients with HER2-positive gastric tumors who might benefit from HER2-targeted therapies. Molecular imaging with ^89^Zr-labeled pertuzumab could, in the right context, improve patient selection for HER2-targeted therapies (22). In addition, we have identified an association between low HER2 membrane staining and high CAV1 protein levels in gastric tumors (27), suggesting a potential role for CAV1 to be used as a complementary biomarker to improve the selection of gastric cancer patients who may benefit from treatment with anti-HER2–targeted therapies.

Molecular imaging studies have shown that tumor accumulation of ^89^Zr-labeled pertuzumab in breast tumors is enhanced in the presence of trastuzumab (24). Our study found similar results in gastric tumors when ^89^Zr-labeled pertuzumab was administered in the presence of an excess of unlabeled trastuzumab. These observations can be explained by trastuzumab induction of HER2 conformational changes that increase pertuzumab affinity for HER2 (42). From the perspective of HER2 endocytosis, our findings suggest that trastuzumab not only induces conformational changes of the HER2 receptor but also enhances pertuzumab intracellular accumulation as a result of trastuzumab-mediated HER2 internalization.

PET imaging using ^89^Zr-labeled full-length antibodies results in unnecessarily high radiation exposure as compared with small molecules labeled with radionuclides with relatively short physical half-lives (e.g., ^18^F). Although pretargeted molecular imaging is a potential alternative to ^89^Zr-labeled antibodies (33), this strategy is dependent on the presence of the target at the cell membrane. HER2 is not an ideal target for pretargeted PET approaches, given that it is a circulating antigen and is internalized through caveolae-mediated endocytic trafficking. Although there has been previous in vitro success for pretargeting of internalizing antibodies in HER2-positive cancer cells, trastuzumab internalization after binding to HER2 results in modest tumor uptake in an in vivo model (33). In our studies, lovastatin increased tumor uptake of ^18^F-radiolabeled Tz in pretargeted imaging approaches using trastuzumab and pertuzumab (Figs. 5 and 6; Supplemental Fig. 8). Further randomized preclinical investigation—using different doses of statin and combinations of statin/antibody—in HER2-positive tumors containing different levels of CAV1 protein and retrospective studies in patients receiving standard doses of statin for concurrent cardiovascular indications while being treated with anti-HER2 antibodies are necessary to determine whether our pharmacological strategy can outperform HER2-targeted imaging/therapy and pretargeted approaches.

## CONCLUSION

Our data on the role of endocytic trafficking in anti-HER2 antibody therapy support the need to consider HER2 membrane availability during patient selection for anti-HER2 therapies. Furthermore, our work indicates that HER2 membrane availability can be modulated with lovastatin to enhance binding of ^89^Zr-labeled pertuzumab in HER2-positive gastric cancer cells characterized by a nonpredominant HER2 membrane staining. These findings are significant as they provide support for pharmacological modulation of CAV1 to improve pretargeted strategies for molecular imaging and therapy of HER2-positive gastric tumors. A limitation of our work is that we could not increase tumor-to-background ratios by extending the injection time between pertuzumab and radiolabeled Tz for time points longer than 24 h. However, our studies provide a foundation for the use of a pharmacological approach to modulate HER2 localization and enhance pertuzumab tumor binding and pretargeted molecular imaging. Future preclinical studies combining pertuzumab plus lovastatin are planned to evaluate whether this combination can improve the therapeutic utility of anti-HER2 targeting in gastric tumors.

## DISCLOSURE

This research was funded in part through the NIH/NCI Cancer Center Support Grant P30 CA008748, NIH U01 CA221046, NIH R01 CA204167, the MSK Geoffrey Beene Cancer Research Center, a Tow Foundation Postdoctoral Fellowship from the MSK Center for Molecular Imaging and Nanotechnology (Patricia Pereira), and a Ruth L. Kirschstein National Research Service Award postdoctoral fellowship (Lukas Carter, NIH F32-EB025050). With regard to this publication, Jason S. Lewis has received research reagents from Genentech and Y. Janjigian has received research funding from Genentech/Roche. No other potential conflict of interest relevant to this article was reported.

## KEY POINTS

**QUESTION:** Does temporal enhancement of HER2 membrane availability, by virtue of caveolin-1 modulation with lovastatin, improve pertuzumab uptake and allow pretargeted molecular imaging of HER2-positive gastric cancer?
**PERTINENT FINDINGS:** Pretreatment of NCI-N87 gastric cancer xenografts with lovastatin enhanced ^89^Zr-labeled pertuzumab tumor uptake, allowing tumor detection as early as 4 h and high-contrast images at 48 h after tracer administration via PET. Temporal enhancement of HER2 membrane availability by lovastatin allowed imaging of cell surface HER2 with transcyclooctene-conjugated antibodies and ^18^F-labeled tetrazine.
**IMPLICATIONS FOR PATIENT CARE:** Our studies support the need to consider HER2 membrane availability during patient selection for anti-HER2 therapies and demonstrate that temporal modulation of membrane HER2 improves pretargeted strategies for molecular imaging and therapy of HER2-positive gastric tumors.
