# Supplementary material for: Temporal Modulation of HER2 Membrane Availability Increases Pertuzumab Uptake and Pretargeted Molecular Imaging of Gastric Tumors
Source: J Nucl Med. 2019 Nov;60(11):1569–78. doi: 10.2967/jnumed.119.225813 (PMC6836866; doi:10.2967/jnumed.119.225813)
Supplement: Supplementary file 1 [file jnm225813SupplementalData.pdf]

Supplemental Data for Pereira et al: Temporal modulation of HER2 endocytosis increases pertuzumab tumor uptake and allows for pretargeted molecular imaging

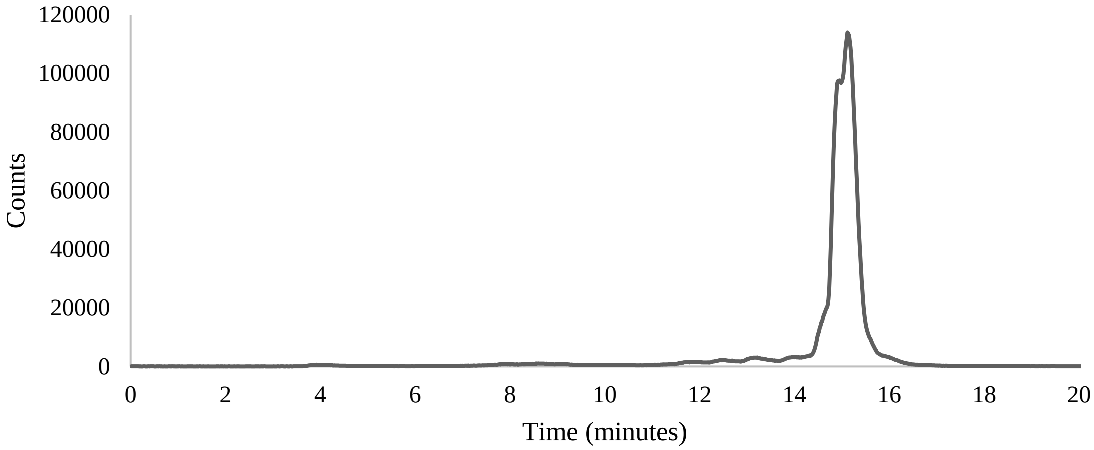

Supplemental Figure 1. Representative quality control (QC) radio-HPLC trace for ^{18}F-labeled Tz, which was obtained with high purity (>98%) and high molar activity (55.5 GBq/µmol).

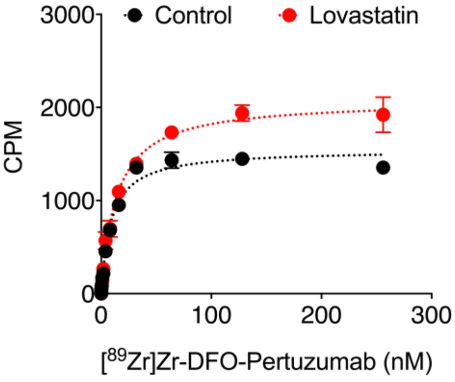

Condition	Bmax (HERs/cell x 10^5)
Control	3.9
Lovastatin	5.4*

Supplemental Figure 2. Binding kinetics of ^{89}Zr-labeled pertuzumab in control and lovastatin-treated NCI-N87 cells. NCI-N87 control and lovastatin-treated cells were incubated with ^{89}Zr-labeled pertuzumab (0 to 256 nM) for 2 h at 4 °C (upper panel). Specific binding of ^{89}Zr-labeled pertuzumab (red or black circles) and non-linear regression curve fit (dotted lines). Data are presented as mean ± S.E.M, n = 3. Binding parameters (lower panel) of ^{89}Zr-labeled pertuzumab to NCI-N87 cells control or treated with lovastatin (*$P < 0.05$ based on a Student's t-test).

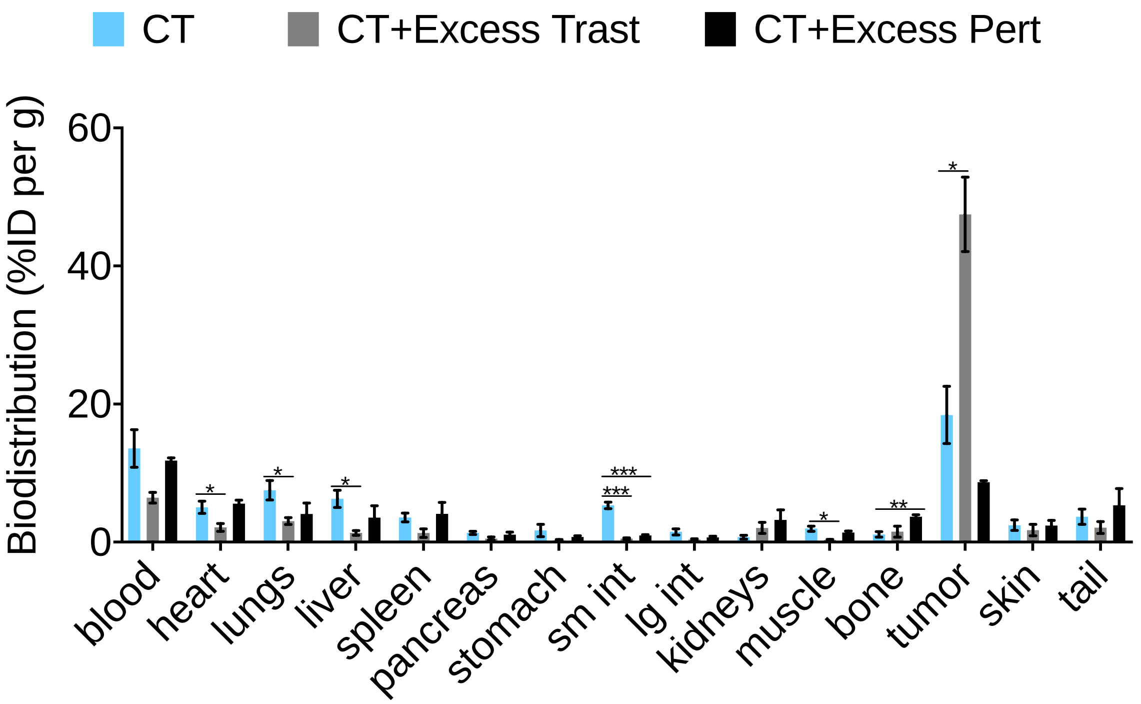

Supplemental Figure 3. Biodistribution in control (CT) athymic nude mice bearing subcutaneous NCI-N87 gastric tumors with and without blocking with unlabeled pertuzumab or trastuzumab. [^{89}Zr]Zr-DFO-pertuzumab (4.44-5.18 MBq, 42-49 μg protein) was administered by tail vein injection. Blocking experiments were performed by administration of ^{89}Zr-labeled pertuzumab in the presence of a 40-fold molar excess of pertuzumab or trastuzumab. Biodistribution studies were performed at 48 h post-injection of ^{89}Zr-labeled pertuzumab. Data represent the mean ± SEM (n = 5 mice per group, * P < 0.05, **P < 0.01, and **P < 0.01 based on a Student's t test). Tumor-to-organ ratios radioactivity are shown in Supplementary Table 2.

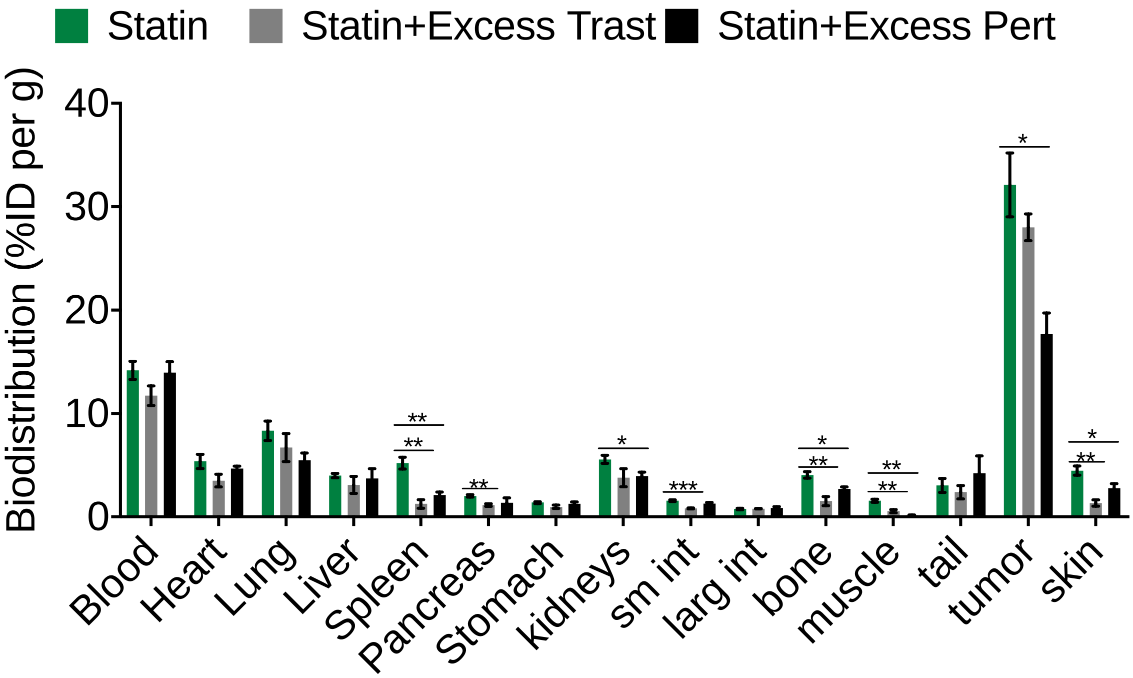

Supplemental Figure 4. Biodistribution in lovastatin-treated athymic nude mice bearing subcutaneous NCI-N87 gastric tumors with and without blocking with unlabeled trastuzumab or pertuzumab. Lovastatin (8.3 mg/kg) was orally administered 12 h prior to and at the same time as the tail vein injection of [^{89}Zr]Zr-DFO-pertuzumab (4.44-5.18 MBq, 42-49 µg protein). Blocking experiments were performed by administration of ^{89}Zr-labeled pertuzumab in the presence of a 40-fold molar excess of trastuzumab or pertuzumab. Biodistribution studies were performed at 48 h post-injection of ^{89}Zr-labeled pertuzumab. Data represent the mean ± SEM, (n = 5 mice per group, *P < 0.05, **P < 0.01, and ***P < 0.001 based on a Student's t test). Tumor-to-organ ratios radioactivity are shown in Supplemental Table 3.

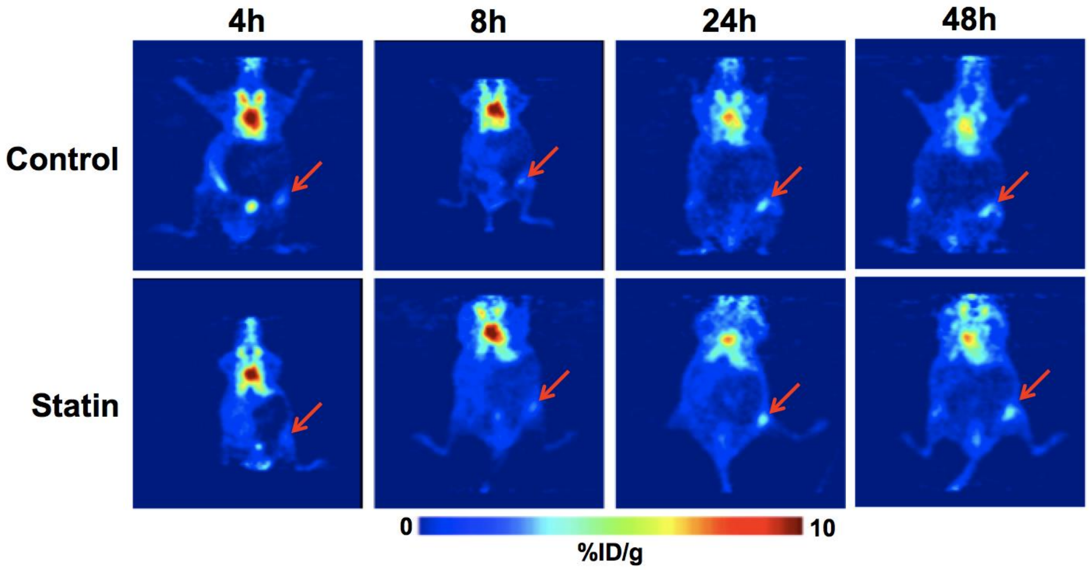

Supplemental Figure 5. Representative coronal PET images of [^{89}Zr]Zr-DFO-pertuzumab in athymic nude mice bearing orthotopic MDA-MB-231 mammary fat pad tumors treated with lovastatin. Lovastatin (8.3 mg/kg of mice) was orally administered 12 h prior and at the same time as the tail vein injection of [^{89}Zr]Zr-DFO-pertuzumab (4.44-5.18 MBq, 42-49 μg protein). Control mice received oral saline instead of lovastatin.

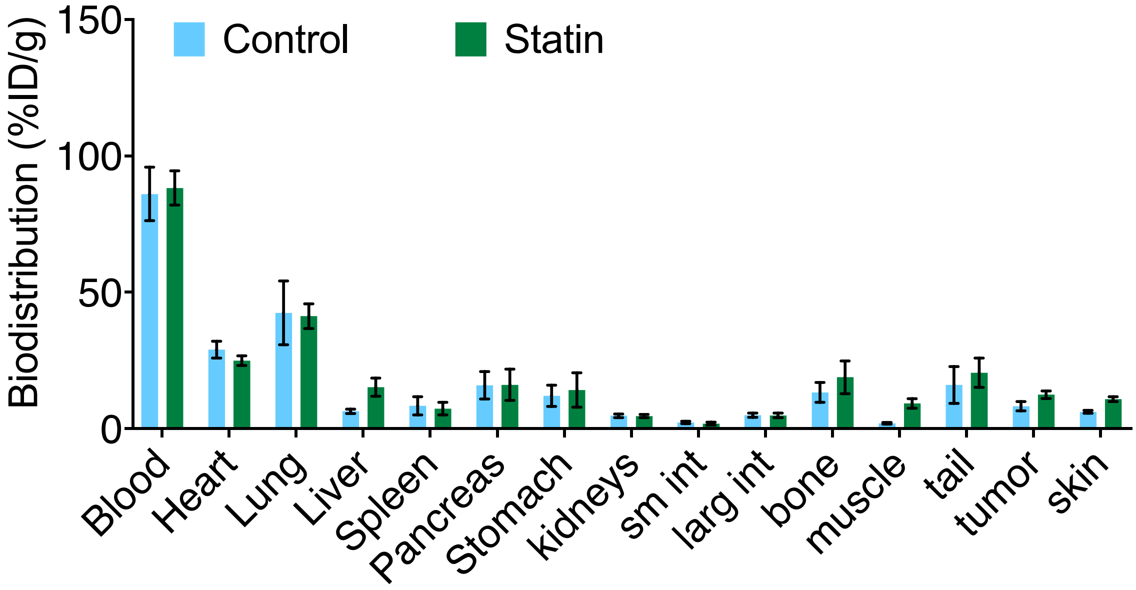

Supplemental Figure 6. Biodistribution of ^{89}Zr-labeled pertuzumab in lovastatin-treated athymic nude mice bearing orthotopic MDA-MB-231 mammary fat pad tumors. Lovastatin (8.3 mg/kg) was orally administered 12 h prior to and at the same time as the tail vein injection of [^{89}Zr]Zr-DFO-pertuzumab (4.44-5.18 MBq, 42-49 µg protein). Biodistribution studies were performed at 48 h post-injection of ^{89}Zr-labeled pertuzumab. Data represent the mean ± SEM, (*n* = 3 mice per group).

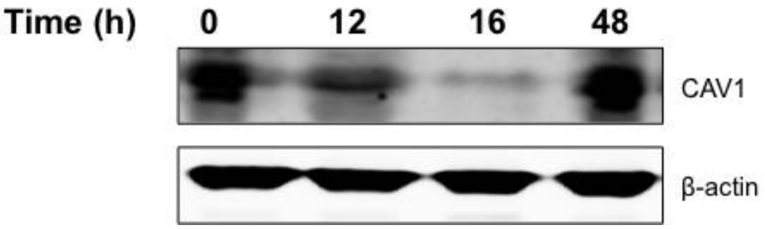

Supplemental Figure 7. Western blot of CAV1 in the total cell lysate of NCI-N87 subcutaneous tumors from athymic nude mice. Lovastatin (8.3 mg/kg) was orally administered twice, with an interval of 12 h between each administration. Tumor lysates were prepared at 0, 12, 16, and 48 h after lovastatin administration and analyzed by Western blot.

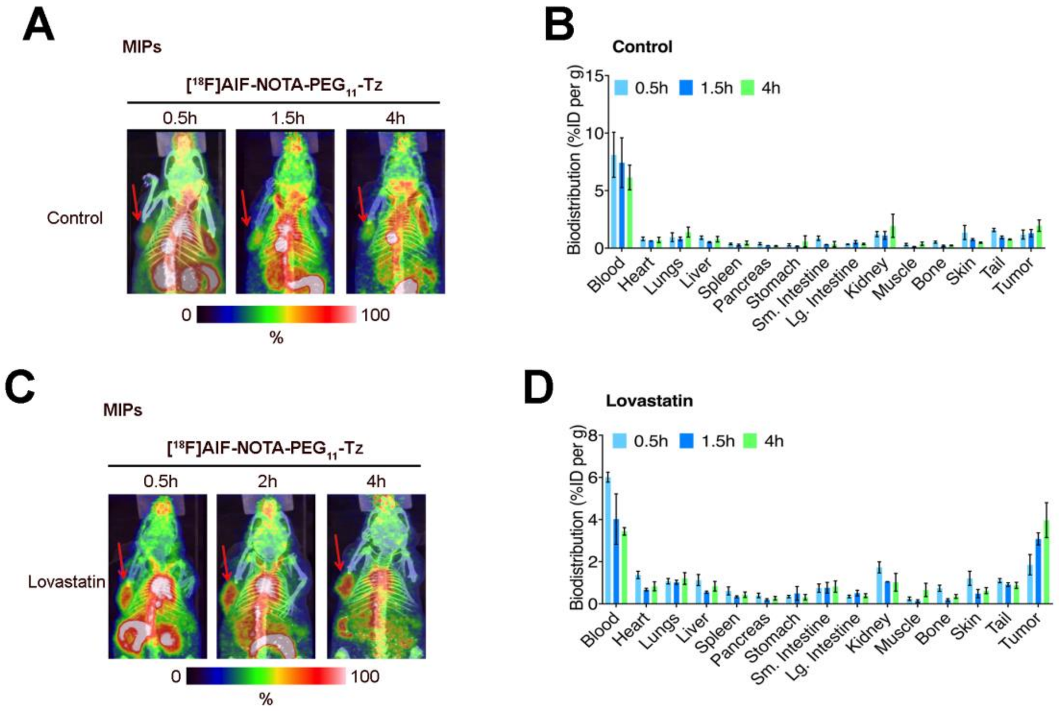

Supplemental Figure 8. (A, C) Representative maximum intensity projection PET images (MIPs) and **(B, D)** biodistribution data at 0.5, 1.5, and 4 h post-injection of [^{18}F]AlF-NOTA-PEG$_{11}$-Tz in athymic nude mice bearing subcutaneous gastric tumors. Lovastatin (8.3 mg/kg) was orally administered 12 h prior to and at the same time as the tail vein injection of trastuzumab-TCO, and then at the same time as the tail vein injection of [^{18}F]AlF-NOTA-PEG$_{11}$-Tz. Mice were given trastuzumab-TCO (0.42 nmol) 24 h prior to injection of the ^{18}F-labeled tracer (10.36-11.1 MBq, 0.83 nmol) via the tail vein. Control (CT) mice received with PBS instead of lovastatin. Biodistribution data represent the mean ± SEM for five mice.

Supplemental Table 1. NCIN87 tumor-to-organ ratios at 48 h after injection of ^{89}Zr-pertuzumab in control and lovastatin-administrated mice.

	Control	Lovastatin
Blood	1.36	2.27
Heart	3.64	5.98
Lung	2.45	3.85
Liver	2.94	8.05
Spleen	5.16	6.17
Pancreas	13.75	15.84
Stomach	10.81	23.25
Kidneys	3.46	5.77
Small intestine	12.41	20.48
Large intestine	25.39	41.55
Bone	9.49	7.92
Muscle	16.37	20.48
Tail	7.50	10.55
Tumor	1.00	1.00
Skin	5.01	7.17

Supplemental Table 2. NCIN87 tumor-to-organ ratios at 48 h after injection of ^{89}Zr-pertuzumab in control mice after blocking with unlabeled trastuzumab or pertuzumab.

	Control	Control + Blocking Trastuzumab	Control + Blocking Pertuzumab
Blood	1.36	7.39	0.73
Heart	3.64	22.39	1.55
Lungs	2.45	15.55	2.14
Liver	2.94	35.41	2.45
Spleen	5.16	36.05	2.11
Pancreas	13.75	90.26	8.06
Stomach	10.81	168.69	11.29
Small Intestine	3.46	91.56	9.05
Large Intestine	12.41	132.13	12.40
Kidneys	25.39	22.87	2.71
Muscle	9.49	155.00	6.28
Bone	16.37	31.23	2.36
Tumor	1.00	1.00	1.00
Skin	7.50	26.96	3.61
Tail	5.01	22.50	1.63

Supplemental Table 3. NCIN87 tumor-to-organ ratios at 48 h post-injection of ^{89}Zr-pertuzumab in statin-treated mice after blocking with unlabeled trastuzumab or pertuzumab.

	Statin	Statin + Blocking Trastuzumab	Statin + Blocking Pertuzumab
Blood	2.27	2.39	1.27
Heart	5.98	7.99	3.79
Lung	3.85	4.17	3.23
Liver	8.05	9.04	4.76
Spleen	6.17	22.21	8.35
Pancreas	15.84	24.40	13.05
Stomach	23.25	28.72	13.89
Kidneys	5.77	7.39	4.48
Small Intestine	20.48	34.20	13.73
Large Intestine	41.55	35.51	20.68
Bone	7.92	18.31	6.52
Muscle	20.48	50.29	109.99
Tail	10.55	11.74	4.19
Tumor	1.00	1.00	1.00
Skin	7.17	20.91	6.42

Supplemental Table 4. NCIN87 tumor-to-organ ratios at 4 h post-injection of [^{18}F]AlF-NOTA-PEG$_{11}$-Tz in control and statin-treated mice administrated with pertuzumab-TCO.

	Control	Lovastatin
Blood	0.13	0.48
Heart	0.41	1.32
Lung	0.31	0.97
Liver	0.57	1.74
Spleen	0.82	2.73
Pancreas	1.08	3.81
Stomach	1.33	4.14
Kidneys	0.28	0.88
Small Intestine	0.53	2.57
Large Intestine	4.68	4.97
Bone	0.70	2.71
Muscle	2.51	4.75
Tail	0.72	2.09
Tumor	1.00	1.00
Skin	1.23	3.87
